# Intolerance of uncertainty in opioid dependency – Relationship with trait anxiety and impulsivity

**DOI:** 10.1371/journal.pone.0181955

**Published:** 2017-07-31

**Authors:** Julia Garami, Paul Haber, Catherine E. Myers, Michael T. Allen, Blazej Misiak, Dorota Frydecka, Ahmed A. Moustafa

**Affiliations:** 1 School of Social Sciences and Psychology, Western Sydney University, Sydney, New South Wales, Australia; 2 National Health and Medical Research Council Centre of Research Excellence in Mental Health and Substance Use, Discipline of Addiction Medicine, Central Clinical School, The University of Sydney, Sydney, New South Wales, Australia; 3 Department of Veterans Affairs, New Jersey Health Care System, East Orange, New Jersey, United States of America; 4 Department of Pharmacology, Physiology & Neuroscience, New Jersey Medical School, Rutgers, The State University of New Jersey, Newark, New Jersey, United States of America; 5 School of Psychological Sciences, University of Northern Colorado, Greeley, Colorado, United States of America; 6 Department and Clinic of Psychiatry, Wroclaw Medical University, Wroclaw, Poland; 7 Department of Genetics, Wroclaw Medical University, Wroclaw, Poland; 8 Marcs Institute for Brain and Behaviour, Western Sydney University, Sydney, New South Wales, Australia; Monash University, AUSTRALIA

## Abstract

Intolerance of uncertainty (IU) is the tendency to interpret ambiguous situations as threatening and having negative consequences, resulting in feelings of distress and anxiety. IU has been linked to a number of anxiety disorders, and anxiety felt in the face of uncertainty may result in maladaptive behaviors such as impulsive decision making. Although there is strong evidence that anxiety and impulsivity are risk factors for addiction, there is a paucity of research examining the role of IU in this disorder. The rate of opioid addiction, in particular, has been rising steadily in recent years, which necessitates deeper understanding of risk factors in order to develop effective prevention and treatment methods. The current study tested for the first time whether opioid-dependent adults are less tolerant of uncertainty compared to a healthy comparison group. Opioid dependent patients undergoing methadone maintenance therapy (n = 114) and healthy comparisons (n = 69) completed the following scales: Intolerance of Uncertainty Scale, the Barrett Impulsivity Scale, and the State Trait Anxiety Inventory. Analysis revealed that these measures were positively correlated with each other and that opioid-dependent patients had significantly higher IU scores. Regression analysis revealed that anxiety mediated the relationship between IU and impulsivity. Hierarchical moderation regression found an interaction between addiction status and impulsivity on IU scores in that the relationship between these variables was only observed in the patient group. Findings suggest that IU is a feature of addiction but does not necessarily play a unique role. Further research is needed to explore the complex relationship between traits and how they may contribute to the development and maintenance of addiction.

## Introduction

In the last 15 years, opioid abuse has grown in the United States by 150% and heroin overdose deaths by 400% [1]. Opioid use and overdose deaths are also steadily on the rise worldwide, including in Europe and Australia [2, 3]. These dramatic increases are largely attributed to the spread of prescription opioid use (and abuse) in demographic groups that have historically been at low risk for addiction, such as women and high income earners [1]. However, the majority of people who use opioid drugs do not become addicted [4, 5]. Accordingly, it is imperative to better understand individual risk factors for drug addiction, such as anxiety, impulsivity, and intolerance of uncertainty (IU).

Drug use has been conceptualized as a maladaptive coping mechanism in which individuals use substances to reduce negative affect elicited by stressful circumstances or distressing emotional states [6]. It has been shown that the reinforcing effect of drugs may be stronger in those who are sensitive to the drug’s stress-alleviating effects [7], and that the reduction of negative affect can occur independently of one’s perception of the “high” of the drug [8]. Reduction of negative affect may be linked to avoidance behaviors, as exaggerated avoidance and slowed extinction have been found in heroin dependent males [9]. Avoidance is a hallmark of anxiety, but this effect was evident even in heroin dependent individuals that were not co-morbid for anxiety.

Anxiety is a common aversive response to stress, and the link between anxiety and substance use disorders has been well documented in the literature [10, 11]. The National Epidemiologic Survey on Alcohol and Related Conditions observed that the comorbidity of substance dependence and anxiety disorders is approximately 25% [12]. Non-clinical anxious traits are also associated with addiction as reported in multiple studies [13–15]. Of the anxiety disorders, generalized anxiety disorder (GAD) has the highest comorbidity rate with drug dependence [10, 12, 16]. GAD is characterized by excessive and uncontrollable worry about potential future events [17].

In turn, IU is defined as a bias to interpret and react negatively to ambiguous situations due to faulty beliefs about uncertainty and its outcomes [18]. These beliefs include notions that uncertainty is unfair, reflects poorly on one’s character, and has unfavorable behavioral consequences [19]. IU is considered to be a transdiagnostic factor across a number of anxiety disorders [20–22]. Studies suggest that IU plays a particularly important role in the development and maintenance of GAD in that IU is instrumental in producing worry, which is the central characteristic of the disorder [23]. However, the effect of IU on worry is not limited to pathological anxiety, as IU has been shown to have a robust association with worry in nonclinical samples [24–26]. IU may further account for high comorbidity rates between GAD and substance abuse in that drug taking may be a way to cope with the negative cognitions and emotions elicited by excessive worry [27].

The existing research on IU and substance use is limited to alcohol use, and has supported the coping model of substance use in that IU predicts drinking alcohol as an avoidance strategy to cope with worry and negative affect [28, 29]. Having negative drinking motivations, such as coping or peer conformity, has been linked to alcohol abuse and developing an alcohol use disorder [30–33]. There is a need to investigate the relationship that IU has with substances other than alcohol, such as opioid drugs, and how IU may impact problematic substance use behaviors that lead to addiction. The profile associated with risk for opioid addiction may be very different, and understanding this is critical as opiate abuse has reached epidemic levels, affects many strata of society, and often has devastating consequences. IU may be a risk factor for developing an addiction if substance abuse is used as a method of coping in the face of unavoidable uncertainty.

Exploring how IU relates to other common addiction features will be useful in furthering our understanding of addiction. It is widely supported that impulsive traits and behaviors are common features of substance use and abuse in both human and animal models [34–38]. While there is evidence linking uncertainty about outcomes to areas of the brain related to impulsivity in drug users [39], there is little research examining impulsivity and IU. Anxiety and impulsivity have traditionally been conceptualized as inverse constructs [40], and the risk aversion hypothesis posits that anxious or worry-prone individuals tend to make fewer risky decisions in order to avoid negative outcomes [41]. It has been shown that people who are prone to worry need to collect more information before making even mundane decisions [42, 43] and take longer to decide as ambiguity increases [44]. As IU is intrinsically linked to anxiety and worry, it could be expected that IU fits the risk aversion hypothesis.

Alternatively, there is evidence for an association between anxiety and heightened impulsivity. An attenuated startle reflex as a measure of the fight/flight response has been observed in highly impulsive participants when presented with distressing images [45]. Research on anxiety disorders has found that novelty-seeking and risk-prone behaviors are characteristic of individuals with social anxiety disorder [46] and obsessive compulsive disorder [47]. Impulsive traits have also been linked to GAD severity and have been shown to significantly predict GAD diagnosis [48]. Additionally, there is evidence that IU may promote impulsive behaviors. Fear of uncertain future events has been associated with heightened psychophysiological reactivity and amplified startle reflexes in anticipation of an unpredictable negative event [49]. GAD and IU have been linked to delay discounting [50] and tendencies to make hasty decisions to alleviate distress in stressful situations [48]. Supporting this, Luhmann et al. [51] found that IU was associated with a preference for an immediate but risky gamble instead of waiting for a gamble with better odds. This behavior was attributed to negative emotions that waiting elicits in those with high IU, in that these participants wanted to quickly end the period of uncertainty regardless of the less favorable outcome. In general, people tend to view hypothetical future rewards as being less certain relative to the length of the delay [52]. It may be that IU magnifies this belief and makes waiting highly unpleasant, leading to quick and less thought-out choices.

There has been no research to date investigating IU and substance addiction. The main purpose of this study was to assess whether opioid-dependent patients experience higher levels of IU compared to a healthy comparison group. We also examined the relationships between IU, impulsivity, and anxiety and whether there are interactions between theses variables on levels of IU. As previous research has found that addiction shows strong associations with anxious behaviors and disorders, the study hypothesized that patients will demonstrate higher IU than a comparison group. Given the evidence of a relationship between impulsivity [45–47], it was also hypothesized that anxiety would mediate the relationship between IU and impulsivity. It is known that addiction is strongly related with impulsivity, however there may be a complex interplay between these factors and IU. Accordingly, we also tested the interaction between addiction and impulsivity/anxiety to ascertain whether they may account for group differences in IU.

## Method

### Participants

The study sample comprised of 183 participants, of which 114 were patients and 69 were healthy comparisons. Written ethics approval was obtained from the Sydney Local Health District human research ethics committee. (Approval number: X16-0356). Participants provided their written informed consent before participating in the study. This consent procedure was approved by the committee. Patients were being treated for heroin addiction by opioid medication (methadone or buprenorphine) at the Drug Health Services and Opioid Treatment Program at the Royal Prince Alfred Hospital in Sydney, Australia. Opioid dependence was confirmed by DSM-IV criteria and urinalysis. The comparison group participants were recruited from psychology students at Western Sydney University and from the wider western Sydney community, through snowballing and advertisements. History of opioid use was an exclusion criterion for the comparison group as assessed by a drug screening questionnaire. All participants completed a demographic questionnaire assessing age, gender, years of education, and efforts were made to match control participants with patients on these variables. Patients also answered clinical questions regarding their age of first opioid use and any secondary drugs of abuse. Patients were considered to be poly-drug users if they reported another drug of concern other than alcohol or other types of opioids. All participants were administered the Intolerance of Uncertainty Scale (IUS), the Barratt Impulsivity Scale (BIS-11), and the State Trait Anxiety Inventory for Adults (STAI).

Eleven participants were not included in the analyses of certain measures due to failure to complete all the questions on the IUS (n = 4), STAI (n = 2), or BIS-11 (n = 5). Data from these participants were included in analyses that did not involve the incomplete questionnaire.

### Measures

#### The Intolerance of Uncertainty Scale (IUS)

The IUS is a 27-item self-report scale measuring negative beliefs about the nature of uncertainty and its consequences [18]. The extent to which the respondent agrees with each item is rated on a 5-point Likert-like scale (1 = not at all characteristic of me to 5 = entirely characteristic of me). The IUS assesses the belief that uncertainty has negative behavioral and self-referent implications, and that uncertainty is unfair and spoils everything. The IUS has been strongly correlated with GAD, worry, anxiety, and depression, and has shown excellent internal consistency (α = .94) and good test-retest reliability (r = .74) [19].

#### The Barratt Impulsivity Scale– 11^th^ revision (BIS-11)

The BIS-11 is a 30-item self-report measure which evaluates impulsivity as a multifactorial behavioral and personality construct [53]. It assesses attentional impulsivity (inability to focus attention/concentrate), motor impulsivity (acting without thinking), and non-planning impulsivity (difficulty planning and thinking carefully about the future). Respondents rate whether a statement reflects the way they act and feel on a 4 point scale (1 = rarely/never to 4 = almost always/always). The BIS-11 is the most widely used measure of impulsiveness, and shows strong internal consistency (α = .83), retest reliability (r = .83), and strong convergent validity with other self-report measures of impulsiveness [37].

#### The State Trait Anxiety Inventory for Adults (STAI)

The STAI is a 40-item self-report questionnaire measuring state anxiety (transient emotions elicited by specific scenarios) and trait anxiety (a relatively consistent predisposition to react to circumstances in an anxious way) [54]. The STAI is comprised of two forms: Form Y-1 assesses state anxiety, and was not administered in this study. Form Y-2 assesses trait anxiety and requires respondents to indicate on a 4-point scale whether a statement reflects how they feel generally (1 = not at all to 4 = very much so). Form Y-2 shows strong internal consistency (α = .89) and retest reliability (r = .88) [55].

### Statistical analyses

The IBM SPSS Statistics version 24 was utilized for the statistical analysis. Independent-samples t-tests were used to assess mean differences in age and years of education between sample groups. The frequency of males and females were analyzed with the chi-square test using Yates’ correction to adjust p values for a 2x2 table. Independent-samples t-tests were used with poly-drug use as the independent variable and scores on the IUS, BIS-11, and STAI as dependent variables. Partial correlations controlling for age, years of education, and gender were employed between all scales. Two hierarchical moderation regression analyses were conducted to determine the unique contribution of addiction status, impulsivity and anxiety variables to IU. A hierarchical regression analysis was also utilized to test for a mediation effect of anxiety on the relationship between impulsivity and IU.

## Results

The patient group was significantly older than the comparison group, t(181) = -2.301, p = .027, and had fewer years of education, t(181) = 9.892, p < .001. There were also significantly more females in the comparison group, χ ^2^ (1) = 9.768, p = .002. Accordingly, these three variables were entered as covariates in the statistical analysis. Overall, patients scored higher on the IUS, BIS-11, and STAI than comparisons. Mean, standard deviations, and percentages for these variables can be found in Table 1.

**Table 1:** Demographic characteristics and mean scores on the IUS, BIS-11, and STAI.

	Comparison	Patients	Statistic
**Age (SD)**	36.58(12.12)	40.27(9.425)	t(182) = -2.301*
**Gender (% female)**	49(71.00%)	54(47.64%)	χ^2^ (1) = 9.768**
**Years of education (SD)**	12.71(1.61)	9.83(2.065)	t(182) = 9.892**
**Age of first heroin use (SD)**		19.00(5.35)	
**Years of addiction (SD)**		21.01(9.86)	
**Poly-drug user (% yes)**		54(49.10%)	
**IUS (SD)**	56.61(16.83)	72.04(24.07)	
**BIS-11 (SD)**	59.19(9.08)	73.55(11.09)	
**STAI(SD)**	35.96(8.20)	48.25(12.33)	

*p < .05.

**p < .01.

Partial correlations found that IUS scores were significantly correlated with BIS-11 scores, r = .2945, p = .003. IUS and BIS-11 scores were also highly correlated with the STAI trait scale at the p < .001 level. Neither age of first use nor length of addiction were significantly correlated with these measures (Table 2).

**Table 2:** Partial correlations between IUS, BIS-11, and STAI trait scores, with gender, age, and education as covariates.

	BIS-11	STAI	Age of first use	Length of addiction
**IUS**	.294*	.546**	.099	-.021
**BIS-11**	—	.492**	.185	-.042
**STAI**		—	.039	.027

*p < .01.

**p < .001.

Mediation regression analysis was conducted on the relationship between questionnaire scores using age, gender, and education as covariates. IUS scores were a significant predictor of STAI scores (b = .305, t(167) = 9.130, p < .001). IUS scores were also a significant predictor of BIS-11 scores (b = .155, t(167) = 4.201, p < .001). When controlling for IUS score, STAI was a significant predictor of BIS-11 scores (b = .533, t(166) = 7.098, p < .001). However, after controlling for STAI scores, IUS was no longer a significant predictor of BIS-11 scores (b = -.008, t(166) = -.191, p = .849). A test of the direct effects using bootstrap estimation indicated that the indirect relationship between IUS and STAI on BIS-11 scores was significant (95% CI [.105-.236]), indicating mediation of STAI on the relationship between IUS and BIS-11 scores.

A three step hierarchical moderated regression tested the independent contributions of demographic variables, group status, and impulsivity to IUS scores (Table 3). Moderation regression was utilized to test the incremental validity of the interaction between groups and impulsivity in explaining group differences in IU. As age, gender, and education were statistically different between groups, these variables were entered into step 1 along with group status. Step 1 of the model accounted for 11.2%% of unique variance (F(4,169) = 5.310, p < .001). After controlling for the variance accounted for by demographic factors, only group status accounted for a significant amount of unique variance in IUS scores (β = .344, t(173) = 3.702, p < .001). BIS-11 scores were introduced in step 2, which accounted for an additional 3.8% of variance (F(1,168) = 7.405, p < .05) in IUS scores. An interaction term between group status and BIS-11 scores was added to step 3 to test for moderation, which yielded a significant 2.1% increase in variance accounted for by the model (F(1,167) = 4.150, p < .05). Group status and BIS-11 scores no longer individually contributed to the variance, indicating a complete moderation. Simple slopes analysis revealed that there was a statistically significant positive relationship between BIS-11 and IUS scores in patients (β = .347, t(173) = 3.451, p = .001) but not comparisons (p>.05).

**Table 3:** Hierarchical moderated regression analysis summary for group status and BIS-11 predicting IUS scores.

Step and predictor variable		F	ΔF	R2	ΔR2	β
**Step 1**		5.310**		.112**		
	**Age**					-.076
	**Gender**					.030
	**Education**					.021
	**Group**					.344**
**Step 2**			7.405*		.038*	
	**Age**					-.031
	**Gender**					.005
	**Education**					.047
	**Group**					.223*
	**BIS-11**					.241*
**Step 3**			4.150*		.021*	
	**Age**					-.071
	**Gender**					.021
	**Education**					.047
	**Group**					-.735
	**BIS-11**					-.045
	**Group x BIS-11**					1.152*

*p < .01.

**p < .001.

A similar second hierarchical moderated regression analyses was conducted with group and STAI scores as independent variables to test the predictive value of addiction and impulsivity on IU (Table 4). Step 1 of the model was identical to the previous hierarchical regression. STAI scores were added to step 2, and accounted for 24.7% of additional variance in IUS scores (F(1,171) = 65.927, p < .001). However, group status no longer independently contributed a significant amount of variance in IU. An interaction between group and STAI scores was added to step 3 to test for a moderation effect, which failed to account for more variance in the model. It appears that when trait anxiety is accounted for in the regression model, addiction status is no longer a strong predictor of IU.

**Table 4:** Hierarchical moderated regression analysis summary for group status and STAI predicting IUS scores.

Step and predictor variable		F	ΔF	R2	ΔR2	β
**Step 1**		5.375**		.111**		
	**Age**					-.059
	**Gender**					.032
	**Education**					.025
	**Group**					.344**
**Step 2**			65.927**		.247**	
	**Age**					.103
	**Gender**					.007
	**Education**					-.020
	**Group**					.018
	**STAI**					.592**
**Step 3**			.044		.000	
	**Age**					1.574
	**Gender**					.105
	**Education**					-.262
	**Group**					.266
	**STAI**					3.888**
	**Group x STAI**					-.210

*p < .01.

**p < .001.

## Discussion

This study sought to add to our understanding of psychological and personality risk factors for opioid drug addiction. One purpose of this study was to assess the relationship between addiction and IU. Our results support the prediction that opioid-dependent patients in the course of methadone maintenance therapy would demonstrate higher IUS scores than a comparison group. Although there were differences in demographic variables between groups, they were not unique predictors of IU. One possible interpretation is that addiction may result from taking drugs regularly in order to cope with an often unpredictable world. Chronic high levels of stress and poor coping mechanisms increase one’s risk of developing a drug addiction. Animal models have shown that stress may permanently alter the structure of the neural reward system in a way that increases the risk of developing addiction [6, 56]. Grupe & Nitschke [57] hypothesized that uncertainty results in hypervigilance, which in drug-dependent populations, may result in enhanced attention to drug-related stimuli and situations thus leading to impulsive behaviors. In addition, stress and anxiety associated with IU can lead to increased drug use and relapse [6, 58].

Alternatively, it is possible that–rather than being risk factors–high IU emerges after the onset of opioid addiction, or even reflect acute drug effects in patients on current methadone therapy. Arguing against this interpretation is the finding in the current study that duration of addiction or poly-drug abuse had no significant relationships with scores on the IUS. This suggests that high levels of IU may be a relatively stable personality trait that pre-date addiction, rather than arising in the wake of opioid exposure/addiction. Only longitudinal studies can definitively answer the question about whether IU antecedes opioid exposure and confer risks for subsequent development of opioid-addiction.

The second aim of the study was to explore the relationship that IU has with impulsivity and anxiety as behavioral and personality factors in addiction, for which prior findings were mixed. IU and trait anxiety in our study were associated with greater impulsivity, which supports research linking dimensions of impulsivity to worry [47, 59, 60], GAD [48, 50, 61], and IU [48, 50, 51]. When these relationships were more closely analyzed with a regression model, the current study found that anxiety mediated the relationship between IU and impulsivity, indicating that erroneous beliefs about uncertainty may not have a unique role in impulsivity. However, mediation analyses assume causality, and due to the cross-sectional design of this study, causal relationships cannot be inferred. If there is a causal relationship between variables, the direction of effects may be the opposite or there may be other variables that have not been accounted for.

We also sought to understand whether the relationship between addiction and IU is moderated by impulsivity. Our results show a significant positive relationship between impulsivity and IU only in the patient group. The relationship between these two individual factors was not found in the comparison group. It appears that there is a particular interplay between impulsivity and IU in opioid-dependent individuals compared to other populations. Impulsivity and IU may additively combine to promote risk for opioid addiction, so that individuals with only one of these traits are less likely to develop addiction. This is supported by Leland et al [39], who found that stimulate drug users had stronger neuronal responses to uncertainty in brain areas associated with impulsive decision making. When the relationships between addiction, anxiety and IU were analyzed, we found that when addiction was accounted for, anxiety was the predominant predictor for IU. That anxiety was such a strong predictor of IU was to be expected given the fundamental link between anxiety and IU, and our results show that this connection is similar between opioid-dependent individuals and healthy comparisons. As our study is the first to investigate IU in relation to addiction, it can be concluded from the current results that there is a complex relationship between addiction and IU that needs to be explored through experimental and longitudinal research.

There are several limitations of the current study that need be taken into account and used to guide future research. Firstly, the battery of psychometric measures was limited and additional ones assessing other traits, personality characteristics, and psychopathologies would have been beneficial in evaluating the validity of the results. Secondly, the self-report methods used here are prone to recall errors or intentional misreporting of sensitive information. The addition of an experimental manipulation for impulsivity, such as a delay discounting task, could help guide the interpretation of our results. Recent work with learning tasks has found that anxiety-vulnerable individuals exhibit enhanced associative learning (e.g., classical eyeblink conditioning) in situations involving some form of uncertainty of stimulus presentation and trial timing [62–64]. Probabilistic category learning tasks have been tested with opioid-dependent individuals [65] who were found to chase reward rather than maintain patterns of responding that are optimal over the long term. Another learning task applicable to addiction in animal models is conditioned place preference in which a location is paired with a reward such as drug administration. Radell et al. [66] found that high IU individuals tended to enter a more rewarding room first and concluded that IU may produce a cognitive bias that results in decision making processes which increase vulnerability to addiction.

Another limitation of this study is the mismatch between comparison and patient groups in age, gender, and education. Previous research using opioid-dependent individuals has shown difficulty in matching these groups closely on education in that these individuals tend to have far fewer years of schooling than their healthy counterparts [9, 65, 67]. While there were significant disparities between groups on these background characteristics, our statistical analysis showed that they did not significantly account for variations in IU. However, there are possible inherent differences between groups such as history of psychiatric illness, socioeconomic status, or negative life events that may have influenced our results. Subsequent studies would benefit from more closely matched control and patient groups.

Overall, the results of this study indicate that opioid-dependent individuals exhibit higher levels of IU than non-drug abusing comparisons, and that the relationship between IU and impulsivity is not apparent in non-drug abusing individuals. This indicates that the relationship between these traits is complex and requires further research to clarify the role that IU plays as a factor in theories of drug addiction.

## Supporting information

S1 Dataset SPSS file containing the full data set used in the study. (SAV)
